# Hydrogen-substituted graphdiyne encapsulated cuprous oxide photocathode for efficient and stable photoelectrochemical water reduction

**DOI:** 10.1038/s41467-022-33445-z

**Published:** 2022-10-01

**Authors:** Xue Zhou, Baihe Fu, Linjuan Li, Zheng Tian, Xiankui Xu, Zihao Wu, Jing Yang, Zhonghai Zhang

**Affiliations:** 1 Shanghai Key Laboratory of Green Chemistry and Chemical Processes, School of Chemistry and Molecular Engineering, East China Normal University, 200241 Shanghai, China; 2 Present Address: Beijing National Laboratory for Molecular Sciences, College of Chemistry and Molecular Engineering, Peking University, 100871 Beijing, China; 3 Present Address: Department of Chemistry, Fudan University, 200438 Shanghai, China

**Keywords:** Photocatalysis, Electrocatalysis, Materials for energy and catalysis, Hydrogen fuel

## Abstract

Photoelectrochemical (PEC) water splitting is an appealing approach for “green” hydrogen generation. The natural p-type semiconductor of Cu_2_O is one of the most promising photocathode candidates for direct hydrogen generation. However, the Cu_2_O-based photocathodes still suffer severe self-photo-corrosion and fast surface electron-hole recombination issues. Herein, we propose a facile in-situ encapsulation strategy to protect Cu_2_O with hydrogen-substituted graphdiyne (HsGDY) and promote water reduction performance. The HsGDY encapsulated Cu_2_O nanowires (HsGDY@Cu_2_O NWs) photocathode demonstrates a high photocurrent density of −12.88 mA cm^−2^ at 0 V versus the reversible hydrogen electrode under 1 sun illumination, approaching to the theoretical value of Cu_2_O. The HsGDY@Cu_2_O NWs photocathode as well as presents excellent stability and contributes an impressive hydrogen generation rate of 218.2 ± 11.3 μmol h^−1^cm^−2^, which value has been further magnified to 861.1 ± 24.8 μmol h^−1^cm^−2^ under illumination of concentrated solar light. The in-situ encapsulation strategy opens an avenue for rational design photocathodes for efficient and stable PEC water reduction.

## Abstract (web summary)

Cu2O-based photocathodes for photoelectrochemical water reduction suffer severe self-photo-corrosion issue. Here the authors report an in-situ encapsulation strategy with hydrogen-substituted graphdiyne to protect Cu2O for water reduction with a photocurrent density of −12.88 mA cm-2.

## Introduction

Large-scale application of hydrogen as clean fuel plays a critical and indispensable role in revolutionizing the current fossil-fuel-based energy system. Hydrogen is as well as an important chemical feedstock and building block, which has been widely utilized in petroleum refining, ammonia synthesis, and core chemicals production. In various sustainable hydrogen production routes, photoelectrochemical (PEC) water splitting is regarded as one of the promising hydrogen generation systems^1–3^, in which, the solar energy collection and water electrolysis are integrated in one device. The PEC device with *p*-type semiconductor as photocathodes (with electron as photo-generated minority charge) can directly generate hydrogen on the surface of photoelectrodes through converting renewable and intermittent solar energy into chemical energy and storing in chemical bonds of hydrogen^4^. Currently, PEC water reduction is well developed with inorganic *p*-type semiconductors, such as silicon^5,6^, metal oxides^7,8^, metal phosphides^9^, and metal sulfides^10,11^. Cuprous oxide (Cu_2_O), a natural *p*-type semiconductor, owns a narrow direct bandgap of 2.0 eV, high theoretical photocurrent density of −14.7 mA cm^−2^ and photoconversion efficiency of 18%, suitable conduction band position (0.7 V higher than the hydrogen evolution potential), and earth-abundance and low cost^12–15^, all of these merits distinguish it to be one of the most promising photocathode candidates for PEC hydrogen generation. Nevertheless, the practical utilization of Cu_2_O-based photocathode for PEC water reduction is still impeded by its intrinsic drawbacks of self-photo-corrosion in aqueous solution and fast surface electron-hole recombination^16,17^, which leads to severe stability and inefficiency issues respectively. To alleviate the above-mentioned limitations, a series of surface protection and modification strategies have been proposed^18,19^. Grätzel and his co-workers in their pioneering work has proposed a multiple-protective-layer strategy with a precision atomic layer deposition method to reach a good stability and high photocathodic current density of −7.6 mA cm^−2^ at potential of 0 V vs RHE (reversible hydrogen electrode) after coupling noble metal electrocatalyst of Pt nanoparticles^12^. Our group have also reported that the stability and PEC performance of Cu_2_O photocathodes have been improved by using carbon, CuO, TiO_2_, and Cu_2_S as multi-functional layers^20–23^. Nevertheless, there is still a big gap between the practical performance and the theoretically maximum photocurrent density on the Cu_2_O-based photocathodes. Therefore, the exploration of “ideal” protective and promotive layer on Cu_2_O photocathode is still a big challenge.

Very recently, an acetylenic carbon-rich framework of hydrogen-substituted graphdiyne (HsGDY) is proposed and synthesized by Li group and reported its excellent electronic conductivity and high stability in aqueous solution^24^. The HsGDY is an extended π-conjugated carbon skeleton comprised of butadiyne linkages and benzene rings. Different from graphene and graphdiyne, the HsGDY has lower atom density, larger pore size with H group in the pores, which would lead to excellent hydrogen ion mobility^24^. Feng and co-workers have even proposed to synthesize HsGDY, named poly(1,3,5-triethynylbenzene) (PTEB) in their paper, on flat copper foil surface and used as free-standing photocathode with a photocurrent density of 10 μA cm^−2^^25,26^. Our group have also prepared the HsGDY on porous copper foam (CF) and reached a high photocurrent density of 1.03 mA cm^−2^ (geometric area)^27^. The HsGDY presents distinguishing advantages of (1) high stability in aqueous solution, (2) robustness under illumination, (3) excellent solid-state charge transfer characteristics, (4) fast hydrogen ion permeation, and (5) facile film-forming processability on copper-based substrates^28,29^, which endow it great potentials as “ideal” candidate to protect and promote Cu_2_O photocathode for efficient and stable PEC applications.

Herein, a photocathode of HsGDY encapsulated Cu_2_O nanowire (HsGDY@Cu_2_O NWs) on three-dimensional porous CF is proposed and prepared. The HsGDY is in-situ generated on the surface of Cu_2_O NWs through a Cu(I) ion mediated Glaser coupling reaction with 1,3,5-triethynylbenzene (TEB) as precursor. The HsGDY@Cu_2_O NWs photocathode demonstrates a high photocurrent density of −12.88 mA cm^−2^ at 0 V vs RHE, approaching to the theoretical photocurrent density of Cu_2_O photocathode under illumination of AM 1.5 G solar light, and a high photoconversion efficiency of 2% is achieved either. In addition, the HsGDY@Cu_2_O NWs photocathode presents excellent stability with only 7.5% loss of photocurrent density after 24 h PEC operation. The efficient PEC performance and high stability enable HsGDY@Cu_2_O NWs photocathode to reach an impressive hydrogen generation rate of 218.2 ± 11.3 μmol h^−1^cm^−2^. Furthermore, the HsGDY@Cu_2_O NWs photocathode has been integrated into a concentrated solar light system, and a high photocurrent density of −50.7 mA cm^−2^ and hydrogen generation rate of 861.1 ± 24.8 μmol h^−1^cm^−2^ are obtained under 10-sun illumination. The HsGDY presents multi-function of protective layer, electron transfer layer, and kinetically catalytic layer. The in-situ encapsulation strategy of dual-functional HsGDY layer opens up an avenue for rational design of Cu_2_O-based photocathodes for efficient and stable PEC water reduction.

## Results

### Preparation and characterization of HsGDY@Cu_2_O NWs

**Fig. 1:**
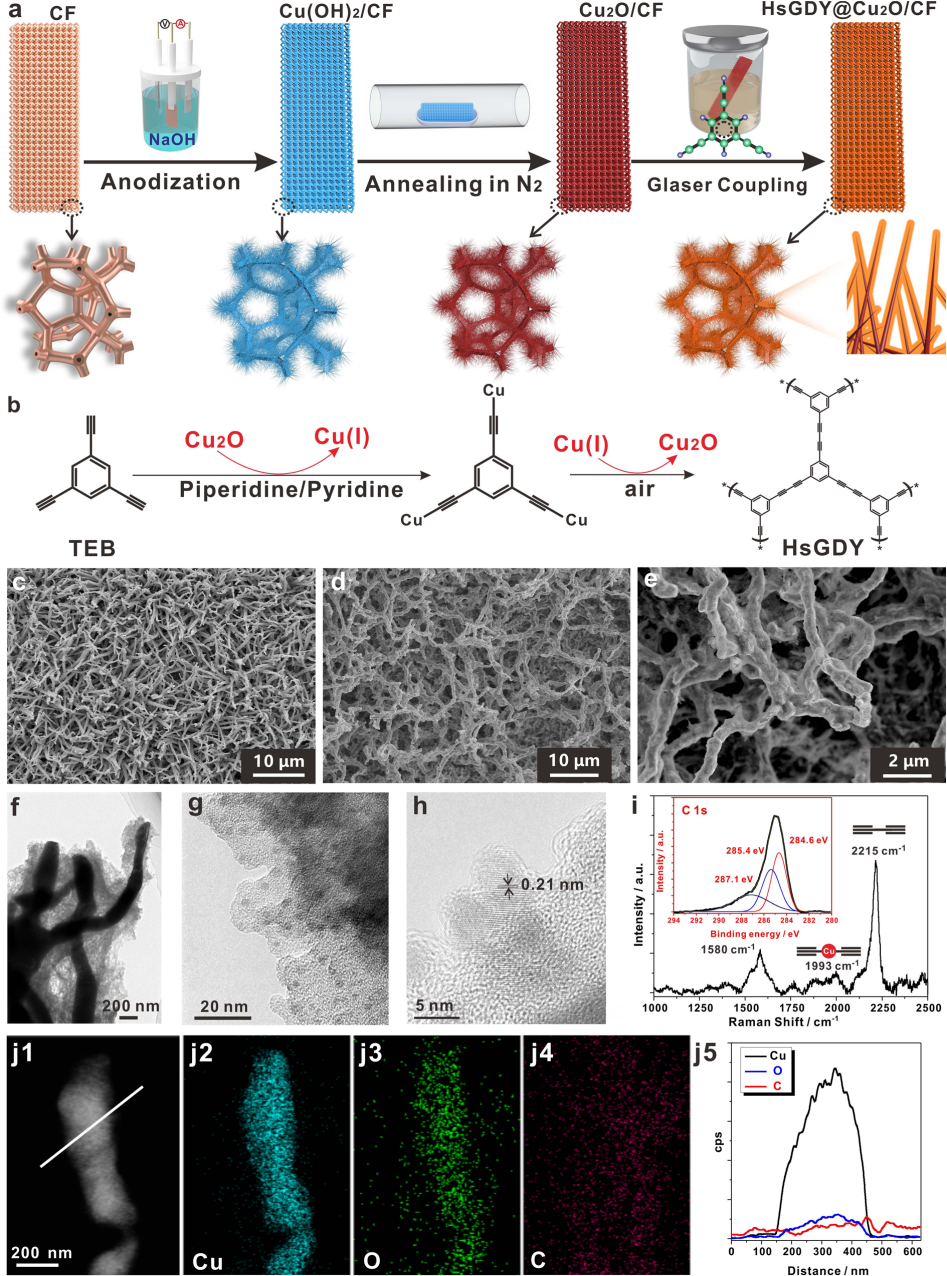
Synthesis and characterizations of HsGDY@Cu_2_O. **a** Schematic illustration of the fabrication of HsGDY@Cu_2_O/CF photocathode; **b** Synthesis of HsGDY with Cu-mediated Glaser coupling reaction; SEM images of **c** Cu_2_O NWs and **d** HsGDY@Cu_2_O NWs; **e** Enlarged SEM image of HsGDY@Cu_2_O NWs; **f** TEM image of HsGDY@Cu_2_O NWs; **g**, **h** HRTEM images of HsGDY on the surface of Cu_2_O NWs; **i** Raman spectrum of HsGDY@Cu_2_O NWs, the inset is the core-level XPS of C 1s of HsGDY@Cu_2_O NWs; **j1** HADDF-STEM image and corresponding EDX mapping images of **j2** Cu, **j3** O, **j4** C elements, and **j5** EDX line scan curves.

The fabrication processes of HsGDY@Cu_2_O NWs on CF are schematically illustrated in Fig. 1a. The CF was rationally selected as substrate of photocathode due to its highly interconnected macroporous structure that brought large surface area and fast electrolyte transfer rate, in addition, the surface metallic Cu can be easily in situ converted to Cu oxides, and the rest metallic Cu skeleton can be directly used as current collector with high electronic conductivity. The HsGDY@Cu_2_O NWs/CF was fabricated through three-step processes of electrochemical- anodization/annealing/Glaser-coupling. The detailed synthesis processes can be found in Method. In brief, the CF was electrochemically anodized in alkali solution to form Cu(OH)_2_ NWs, which then underwent an annealing process in nitrogen atmosphere and converted to Cu_2_O NWs, finally, the Cu_2_O NWs was conformally coated with microporous HsGDY layers. As presented in Fig. 1b, Glaser coupling reaction occurred with 1,3,5-triethynylbenzene (TEB) as precursor, piperidine as ligand, pyridine as solvent, and Cu(I) ion as catalyst (slightly dissolved from Cu_2_O in polar solvent), and the HsGDY layer was generated with giant two-dimensional network structure that comprised large organic hexatomic rings as the construction unit and alternating-distributed benzene ring and alkynyl as sub-units.

The micro-morphologies of Cu_2_O NWs and HsGDY@Cu_2_O NWs were characterized from scanning electron microscope (SEM) and transmission electron microscope (TEM). As depicted in Fig. 1c, the Cu_2_O sample showed a tapered off nanowire structure with length of several micrometers. For HsGDY@Cu_2_O NWs sample, as presented in Fig. 1d, e, the nanowire structures were still well maintained with much rougher and gossamer-like surface, indicated the coating of HsGDY on the surface of Cu_2_O NWs. The elemental compositions of HsGDY@Cu_2_O NWs were detected from energy dispersive X-ray spectrum (EDS) (Supplementary Fig. 1) and only Cu, O, and C elements were identified. The TEM image in Fig. 1f further revealed the conformal nanowire structure with clear HsGDY layer covered on the surface of Cu_2_O NWs. In addition, the high-resolution TEM (HRTEM) image in Fig. 1g uncovered stacked sheets of HsGDY. Surprisingly, some small dots with size of ~3 nm were determined to be embedded on HsGDY sheet. Closer observation as shown in Fig. 1h, clear lattice with interplanar spacing of 0.21 nm can be ascribed to (200) plane of Cu_2_O. The formation of Cu_2_O nanodots was probably due to the micro-dissociation of Cu_2_O NWs in polar solvents, which lead to the production of Cu(I) ions and mediated the Glaser coupling reaction, then, after the formation of HsGDY, the Cu(I) ions converted to the state of Cu_2_O and accumulated in HsGDY sheets.

The crystal structure of HsGDY@Cu_2_O NWs was revealed from X-ray diffraction (XRD) pattern (Supplementary Fig. 2), and all diffraction peaks can be ascribed to metallic Cu and Cu_2_O. No diffraction peak of HsGDY was detected, which implied amorphous nature of HsGDY. To further indicate the presence of HsGDY framework, Raman spectrum and x-ray photoelectron spectrum (XPS) of HsGDY@Cu_2_O NWs were measured and presented in Fig. 1i. For Raman analysis, the strongest peak at 2215 cm^−1^ can be ascribed to the typical acetylenic bond (C ≡ C) stretching vibration, and the peaks in 1500–1650 cm^−1^ can be assigned to the stretching vibrations of aromatic ring with a strong G-band peak located at 1580 cm^−1^^25^, which suggested that the samples possessed abundant aromatic rings. Except these typical Raman peaks of HsGDY, an unusual peak at 1993 cm^−1^ with relative weak intensity has been observed, which can be ascribed to the formation of Cu-metalated C ≡ C stretching^30^. The XPS survey revealed that the HsGDY@Cu_2_O NWs contained only elemental copper, O, and carbon (Supplementary Fig. 3), in addition, compared with pristine Cu_2_O NWs, the HsGDY@Cu_2_O NWs presented much higher intensity of carbon element. The core-level XPS C 1 s of HsGDY@Cu_2_O NWs was depicted in the inset of Fig. 1i, and the deconvoluted subpeaks at 284.6 eV and 285.4 eV can be ascribed to C = C (sp^2^) and C ≡ C (sp), respectively^30^. The area ratio of sp^2^ and *sp* carbon was close to 1:1, in accordance with the structure of HsGDY, and thus provided further robust evidence for the formation of HsGDY. Except the two main subpeaks, the peak at 287.1 eV can be assigned to C–O. However, compared to binding energy of 287.5 eV of C–O bond obtained from individual HsGDY^24^, the negative shift of binding energy implied the potential electron transfer from Cu_2_O to HsGDY. To further reveal the elemental distribution on single HsGDY@Cu_2_O nanowire, high-angle annular dark-field scanning transmission electron microscopy (HADDF-STEM) image (Fig. 1j1) and corresponding EDS elemental mapping images were measured and revealed uniform distribution of Cu (Fig. 1j2), O (Fig. 1j3), and C (Fig. 1j4) elements on nanowire. In addition, the line scan EDS curves (Fig. 1j5) presented obvious expansion of carbon element distribution on single HsGDY@Cu_2_O nanowire, which further indicated encapsulation of Cu_2_O nanowire with HsGDY. All of the above experimental results indicated intense interaction between Cu_2_O and HsGDY. The unique encapsulation structure of HsGDY@Cu_2_O NWs portended promising charge transfer activity and excellent PEC performance can be expected.

### The PEC performance of HsGDY@Cu_2_O NWs

**Fig. 2:**
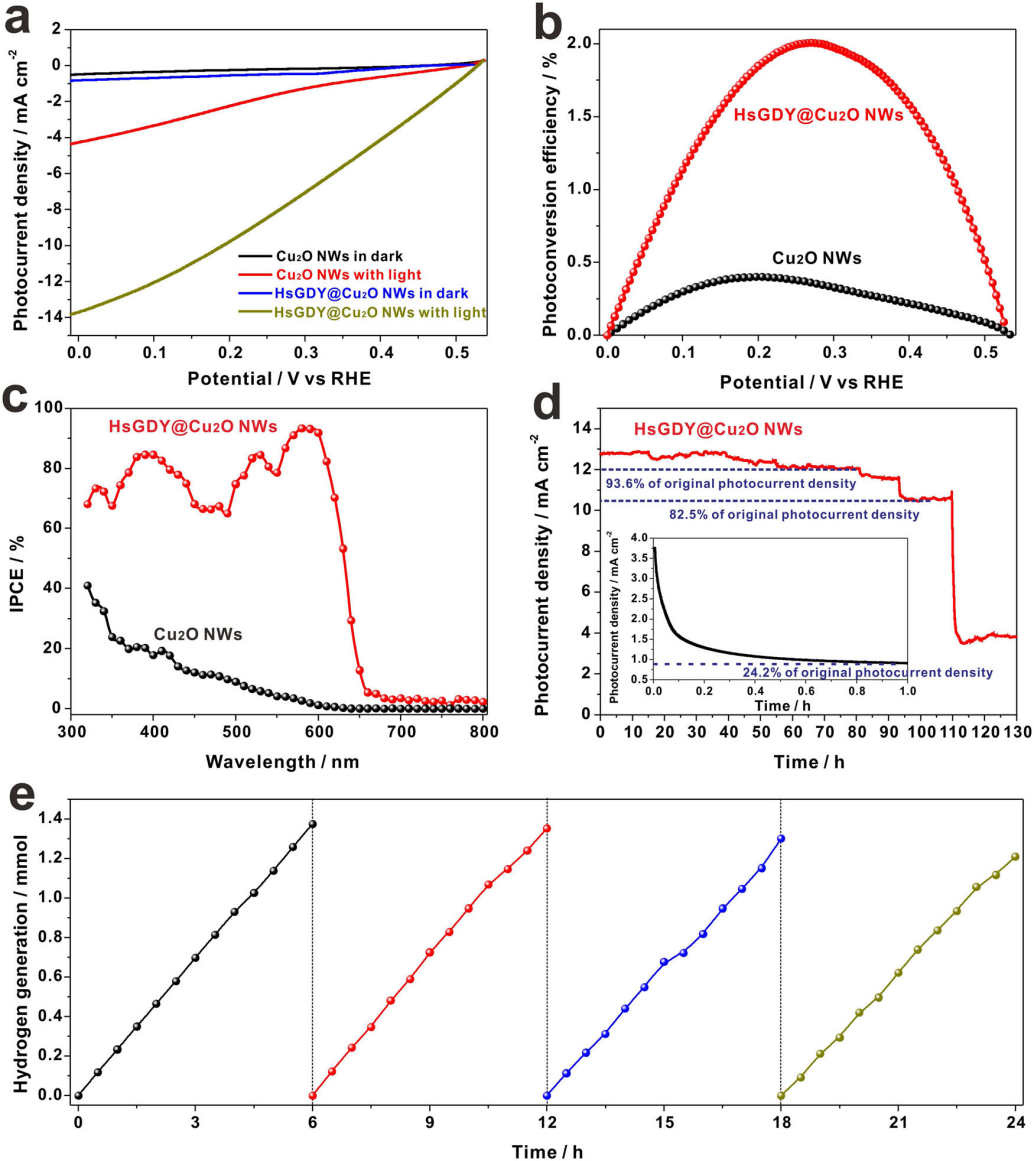
PEC water reduction on HsGDY@Cu_2_O NWs photocathode. **a** LSV plots of Cu_2_O NWs and HsGDY@Cu_2_O NWs in dark and under illumination of simulated solar light (AM 1.5 G, 100 mW cm^−2^), recorded with scan rate of 5 mV s^−1^ in 0.1 M Na_2_SO_4_ solution with pH = 4.9; **b** Photoconversion efficiency of Cu_2_O NWs and HsGDY@Cu_2_O NWs; **c** IPCE plots of Cu_2_O NWs and HsGDY@Cu_2_O NWs under a constant potential of 0 V vs RHE in wavelength region from 300 nm to 800 nm; **d** Stability measurement of HsGDY@Cu_2_O NWs at potential of 0 V vs RHE, the inset is the chronoamperometric curve on Cu_2_O NWs; **e** Hydrogen generation on HsGDY@Cu_2_O NWs/CF photocathodes at potential of 0 V vs RHE under illumination of simulated solar light (100 mW cm^−2^, AM 1.5 G).

The PEC water reduction performance of HsGDY@Cu_2_O NWs was evaluated by linear sweep voltammetry (LSV) method under illumination of simulated solar light (AM 1.5 G, 100 mW cm^−2^). The Glaser coupling reaction durations have been optimized to maximize the PEC performance of HsGDY@Cu_2_O NWs with different HsGDY thickness (Supplementary Fig. 4). As presented in Fig. 2a, both photocathodes under dark conditions did not show measurable HER activity in the potential window. While, under illumination, the optimized HsGDY@Cu_2_O NWs/CF photocathode showed a high photocurrent density of −12.88 mA cm^−2^ at 0 V vs RHE, much higher than the photocurrent density of −4.2 mA cm^−2^ on pristine Cu_2_O NWs/CF photocathode. The high photocurrent density value was closely approaching to the theorical maximum photocurrent density of Cu_2_O-based photocathodes^12^. The LSV plot of HsGDY@Cu_2_O NWs/CF photocathode under illumination of chopped light was also measured and presented fast light-dark current conversion with similar values (Supplementary Fig. 5). In addition, the electrochemical active surface area of HsGDY/Cu_2_O NWs were also estimated through Helmholtz double layer capacitance measurements^31^. The electrochemical active surface area can be measured to be 1.38 ± 0.63 cm^2^ (Supplementary Fig. 6), therefore, the intrinsic photocurrent density of HsGDY@Cu_2_O NWs/CF can be converted to be 9.33 ± 2.92 mA cm^−2^, which value indicated the efficient intrinsic PEC activity of HsGDY@Cu_2_O NWs. The HsGDY on pristine CF was also prepared and the corresponding PEC and optical properties have been measured (Supplementary Fig. S7). The relative low PEC performance further implied the decisive role of charge transfer of HsGDY layer.

The photoconversion efficiencies of Cu_2_O NWs and HsGDY@Cu_2_O NWs photocathodes were calculated using equation of ***η*** = *J*_Pmax_*V*_Pmax_/*P*_in_, where *J*_Pmax_ and *V*_Pmax_ are the current density (mW cm^−2^) and photovoltage (V vs RHE) at the maximum power point and *P*_in_ (mW cm^−2^) is the incoming light flux (100 mW cm^−2^)^32^. The plots of photoconversion efficiency vs potential vs RHE were presented in Fig. 2b, where, the Cu_2_O NWs exhibited an optimal conversion efficiency of 0.40% at 0.197 V, while, the HsGDY@Cu_2_O NWs achieved a much higher optimal conversion efficiency of 2.0% at 0.275 V.

To better elucidate the enhancement of PEC performance after HsGDY encapsulated Cu_2_O NWs, incident photon-to-current-conversion efficiency (IPCE) measurements were conducted on Cu_2_O NWs and HsGDY@Cu_2_O NWs. The IPCE value was estimated and calculated with following equation:^33^

1 $${{{\rm{IPCE}}}}(\%)=1240{{{\rm{I}}}}/\lambda {{{{\rm{P}}}}}_{{{{\rm{light}}}}}\times 100\%$$

where *I* is the photocurrent density (mA cm^−2^), *P*_light_ is the incident light irradiance (mW cm^−2^), and *λ* is the incident light wavelength (nm). As presented in Fig. 2c, both Cu_2_O NWs and HsGDY@Cu_2_O NWs showed PEC response threshold at ~650 nm, whereas, the HsGDY@Cu_2_O NWs exhibited considerably enhanced PEC performance in broad wavelength region from 650 nm to 320 nm. The photocurrent density under illumination of solar light can be integrated from the IPCE values and solar irradiance and the corresponding integrated photocurrent density was 12.82 mA cm^−2^ (Supplementary Fig. 8), which value was close to photocurrent density of 12.88 mA cm^−2^ under illumination of simulated solar light with AM 1.5 G filter. The insignificant variation can be ascribed to the slight difference of irradiance spectra of simulated solar light and solar light. More interestingly, the IPCE value on HsGDY@Cu_2_O NWs increased rapidly with decreasing wavelength in a small region between 650 nm to 570 nm, and reached the highest value of 93.3% at wavelength of 580 nm. In addition, IPCE plots on HsGDY@Cu_2_O NWs at different applied potentials have been recorded (Supplementary Fig. 9). The HsGDY@Cu_2_O NWs at applied potential of +0.4 V vs RHE presented slow IPCE increase tendency, which can be ascribed to the slow charge transfer due to the relative positive potential. While, at applied potential of −0.4 V vs RHE, the IPCE plot displayed similar increase tendency with that at 0 V vs RHE with higher IPCE values. The fast IPCE increase indicated that the coupling of HsGDY can effectively promote the charge separation on Cu_2_O NWs, and greatly increased the probability of photoelectrons generation. In addition, a unique multi-band shape of the IPCE curve of HsGDY@Cu_2_O NWs can be observed. This phenomenon may be ascribed to the photon competition between HsGDY and Cu_2_O, and thus presented optical absorption “filter” property at special wavelength.

Long-term PEC stability is crucial for practical applications of photoelectrodes, especially for Cu_2_O-based photocathodes. The PEC stability of Cu_2_O NWs and HsGDY@Cu_2_O NWs were evaluated with a steady-state chronoamperometry, and the results were presented in Fig. 2d. The photocurrent on Cu_2_O NWs rapidly decayed and remained 24.2% to its initial photocurrent after 1 h illumination (the inset in Fig. 2d). Nevertheless, the photocurrent on HsGDY@Cu_2_O NWs maintained 93.6% of its original value after continuous illumination for 80 h, and 82.5% of original photocurrent was remained after 100 h measurements. The encapsulation strategy with HsGDY indeed protected the Cu_2_O NWs from self-photo-corrosion. With longer stability measurements, after 120 h duration, the photocurrent decreased to ~3.5 mA cm^−2^, which can be ascribed to the partial broken of the HsGDY@Cu_2_O layer due to the violently producing hydrogen bubbles (Supplementary Fig. 10). The chemical compositions after long-term measurements were as well as characterized, and the emergence of metallic Cu species also suggested the exposure of copper foam surface after falling off the HsGDY@Cu_2_O NWs (Supplementary Fig. 11).

Furthermore, the practical application of PEC water reduction for hydrogen generation on HsGDY@Cu_2_O NWs photocathode were evaluated and presented in Fig. 2e. The hydrogen generation experiments were repeated for 4 cycles with one cycle of 6 h, and an impressive generation rate of 218.2 ± 11.3 μmol h^−1^ cm^−2^ was achieved. The Faraday efficiency for PEC hydrogen generation was also calculated to be 96.1 ± 2.3% during the entire water reduction process, which indicated that almost of the photogenerated carriers virtually participated into the process of hydrogen generation. We have electrodeposited Pt nanoparticles on the surface of HsGDY, and no further increase of the photocurrent density can be recorded (Supplementary Fig. 12), which implied that the HsGDY worked as kinetically active layer for hydrogen generation. The acetylene units (-C ≡ C-) can be well regarded as efficient active sites for PEC hydrogen generation^26,34,35^. Up to now, the excellent PEC performance of HsGDY@Cu_2_O NWs has proposed that the encapsulation strategy with HsGDY on Cu_2_O NWs was a very successful attempt, and to the best of our knowledge, the HsGDY@Cu_2_O NWs contributed the highest photocurrent density value ever reported on Cu_2_O materials with/without using of noble metal co-catalysts (Supplementary Fig. 13 and Supplementary Table 1). The HsGDY presented multi-function of protective layer, electron transfer layer, and kinetically catalytic layer, all of these roles contributed to the efficient PEC water reduction for hydrogen generation.

### Mechanism of enhanced PEC performance on HsGDY@Cu_2_O NWs

**Fig. 3:**
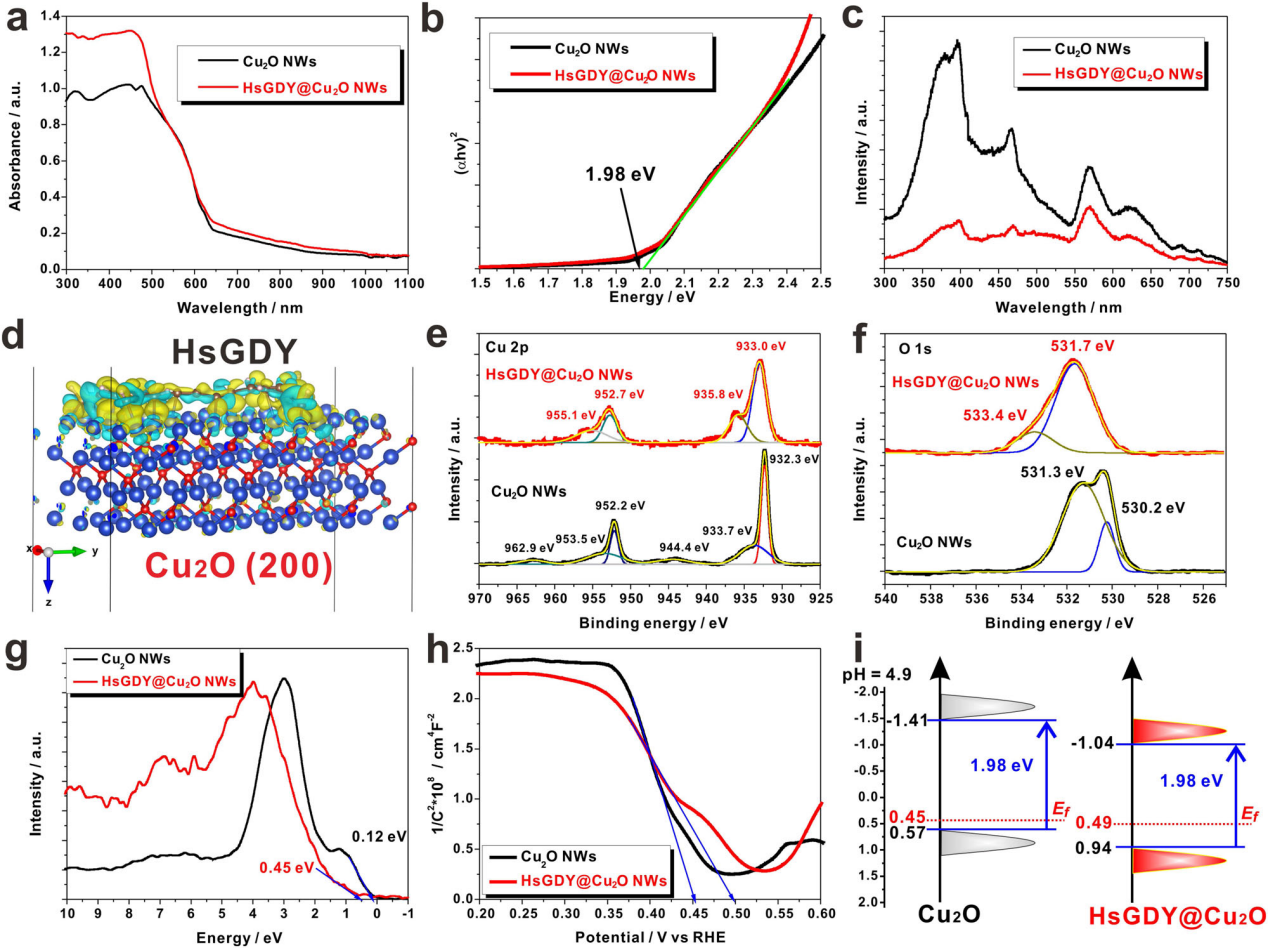
Multiple characterizations of interaction between HsGDY and Cu_2_O NWs. **a** Optical absorption spectra of Cu_2_O NWs and HsGDY@Cu_2_O NWs; **b** Tauc plots of Cu_2_O NWs and HsGDY@Cu_2_O NWs; **c** Fluorescence spectra of Cu_2_O NWs and HsGDY@Cu_2_O NWs; **d** DFT calculation of difference charge density on the interface of HsGDY and Cu_2_O; Core-level XPS of **e** Cu 2p and **f** O 1 s of Cu_2_O NWs and HsGDY@Cu_2_O NWs; **g** valence band XPS of Cu_2_O NWs and HsGDY@Cu_2_O NWs; **h** Mott-Schottky plots of Cu_2_O NWs and HsGDY@Cu_2_O NWs; **i** Band energy structure diagrams of Cu_2_O NWs and HsGDY@Cu_2_O NWs.

To deeply reveal the mechanism of significantly enhanced PEC performance on HsGDY@Cu_2_O NWs, a series of experiments and calculation were conducted. The optical absorption property of Cu_2_O NWs and HsGDY@Cu_2_O NWs were measured and the UV-Vis-NIR diffuse reflection spectra were presented in Fig. 3a. Both samples showed similar absorption threshold at ~640 nm, but the HsGDY@Cu_2_O NWs depicted higher absorbance in the range of 300–500 nm. The band gap values of these two materials were also estimated from the corresponding Tauc plots. As shown in Fig. 3b, both samples presented the same band gap value of 1.98 eV. Although the encapsulation Cu_2_O NWs with HsGDY did not change the band gap, the absorbance was increased in a wide spectral range, which was beneficial to the improvement of PEC performance. In addition, the fluorescence emission spectra of Cu_2_O NWs and HsGDY@Cu_2_O NWs have been recorded and presented in Fig. 3c. The Cu_2_O NWs showed few sharp and intensive emission fluorescence peaks, while, after encapsulation with HsGDY, the fluorescence emission has been severely quenched, which helped to gain more insights into the potential charge carrier transfer between HsGDY and Cu_2_O NWs.

The potential electron transfer direction between Cu_2_O NWs and HsGDY has been predicted by density functional theory (DFT) calculations. All calculations were performed using CP2K quantum chemistry software package with Perdew–Burke–Ernzerhof (PBE) parametrization of generalized gradient approximation to describe the exchange-correlation part in Hamiltonian^36^. The HsGDY@Cu_2_O NWs heterostructures were constructed by matching the layer HsGDY on the Cu_2_O (200) surface based on the experimental results. As shown in Fig. 3d, clear spatial charge redistributions on the interface of HsGDY and Cu_2_O was depicted, and based on the Mulliken population analysis, each Cu_2_O donated 0.23 electron to adsorbed HsGDY. The DFT calculation results indicated that photo-generated electrons in Cu_2_O can be expected to efficiently transfer to HsGDY, and thus facilitated the charge separation.

To further verify the charge transfer and chemical states of Cu and O atoms, core-level XPS of Cu 2*p* and O 1*s* in pristine Cu_2_O and in HsGDY@Cu_2_O were measured and are presented in Fig. 3e, f respectively. For Cu_2_O NWs, the binding energy of at 932.3 eV and 952.2 eV can be ascribed to Cu(I) 2*p*_3/2_ and Cu(I) 2*p*_1/2_, and the broad and weak peaks at 933.7 eV and 953.5 eV as well as the satellite peaks at 944.4 eV and 962.9 eV indicated the existence of small amount of CuO on the surface of Cu_2_O due to the inevitable oxidation in air^37^. For HsGDY@Cu_2_O NWs, the binding energy of Cu(I) 2*p*_3/2_ and Cu(I) 2*p*_1/2_ positively shifted to 933.0 and 952.7 eV, which implied the decrease of electron cloud density on Cu atoms due to the charge transfer from Cu_2_O to HsGDY. In addition, the core-level O 1 s of Cu_2_O presented two peaks at the binding energies of 530.2 eV and 531.3 eV, which can be attributed to the oxygen in Cu_2_O lattice and oxygen associated with an oxygen vacancy environment^38^. After encapsulated by HsGDY, the strong peak at 531.7 eV indicated the formation of Cu–O–H structure^39^, which implied that the dominated connection between the hydrogen in HsGDY and the oxygen in Cu_2_O. Except the oxygen in lattice, the weak peak in 533.4 eV can be ascribed to the formation of C–O bond^40^. Therefore, the XPS results further validated the prediction about efficient charge transfer from Cu_2_O to HsGDY from the DFT calculation.

For efficient water reduction on photocathode, except the virtues of proper bandgap and fast charge transfer, the suitable band positions are as well as essential. The valence band-XPS (VB-XPS) measurements were conducted on Cu_2_O NWs and HsGDY@Cu_2_O NWs and are presented in Fig. 3g. The Cu_2_O NWs and HsGDY@Cu_2_O NWs showed distances from VB edge level of 0.12 eV and 0.45 eV corresponding to their Fermi level (E_f_) respectively. In addition, Mott−Schottky (M-S) plots were also recorded to determine the flat band potentials, which would reflect the differences between E_f_ and water-reduction potential. As presented in Fig. 3h, the flat band potentials on Cu_2_O NWs and HsGDY@Cu_2_O NWs were determined to be 0.45 V and 0.49 V respectively. Finally, based on the band gap values from Fig. 3b and the results from VB-XPS (Fig. 3g) and M-S plots (Fig. 3h), the band energy structure diagrams of Cu_2_O NWs and HsGDY@Cu_2_O NWs were illustrated in Fig. 3i, where the enlarged distance between E_f_ and VB in HsGDY@Cu_2_O further revealed higher electron density than that in Cu_2_O. This result can be ascribed to the π-electron delocalization and effective electron transfer from Cu_2_O to HsGDY, and the corresponding results of lower electronic resistance have as well as been obtained from electrochemical impedance spectra (Supplementary Fig. 14).

### PEC water reduction under concentrated solar light

**Fig. 4:**
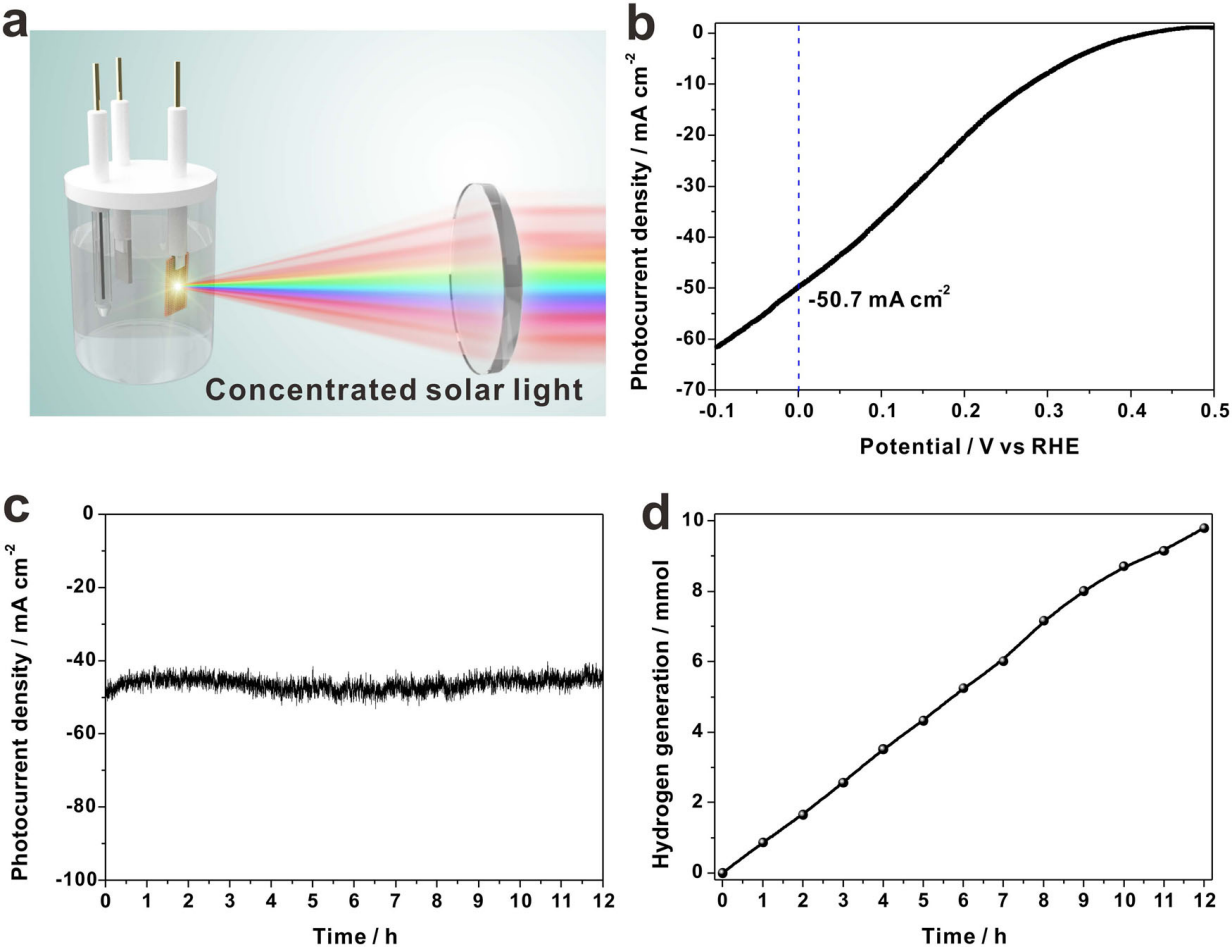
PEC water reduction on HsGDY@Cu_2_O NWs with concentrated solar light. **a** Schematic illustration of PEC water reduction device with concentrated solar light illumination; **b** LSV plot of HsGDY@Cu_2_O NWs under illumination of 10-sun (AM 1.5 G, 1000 mW cm^−2^), recorded with scan rate of 5 mV s^−1^ in 0.1 M Na_2_SO_4_ solution with pH = 4.9; **c** Long-term PEC stability test on HsGDY@Cu_2_O NWs photocathodes under illumination of 10-sun; **d** Hydrogen generation on HsGDY@Cu_2_O NWs/CF photocathode at potential of 0 V vs RHE under illumination of 10-sun.

The PEC water reduction under illumination with high light intensity is a facile and promising way to accelerate hydrogen generation rate in limited time^41,42^. The HsGDY@Cu_2_O NWs based photocathode was integrated into a concentrated solar light system (Fig. 4a), established through a simple Fresnel lens, and a high photocurrent density of −50.7 mA cm^−2^ at 0 V vs RHE was obtained under 10-sun illumination (Fig. 4b). The Fresnel lens was comprised with cheap plastics, which was beneficial for its large-scale application in low-cost. Long-term PEC stability on HsGDY@Cu_2_O NWs were evaluated with a steady-state chronoamperometry under illumination of 10-sun, and the results were presented in Fig. 4c. The high photocurrent density did not show significantly decay after 12 h test. The fast hydrogen generation was also recorded for practical application of PEC water reduction under 10-sun illumination (Fig. 4d), and the hydrogen generation rate reached 861.1 ± 24.8 μmol h^−1^ cm^−2^.

## Discussion

In summary, an encapsulation strategy was proposed to prepare HsGDY@Cu_2_O NWs photocathode. The HsGDY was selected with dual-functions of protection and charge transfer layer. The rational designed HsGDY@Cu_2_O NWs photocathode demonstrated a high photocurrent density and high stability with impressive hydrogen generation rate of 218.2 ± 11.3 μmol h^−1^cm^−2^. To further increase the hydrogen generation rate in limited time, the HsGDY@Cu_2_O NWs photocathode was integrated into a concentrated solar light system, and obtained a high photocurrent density of −50.7 mA cm^−2^ and hydrogen generation rate of 861.1 ± 24.8 μmol h^−1^cm^−2^. Our study presented a straightforward approach for achieving highly efficient and stable Cu_2_O NWs-based photocathode. We believe the encapsulation strategy will open an avenue of rational designing efficient photoelectrodes for PEC water splitting.

## Methods

### Chemicals and materials

A 1-mm-thick copper foam supplied by Suzhou Taili New Energy Materials Co., Ltd. (China), was cut into pieces of 50 × 10 mm^2^. Pyridine (99.5%), methanol (99.9%), dichloromethane (AR), sodium hydroxide, potassium dihydrogen phosphate, and sodium sulfate were purchased from Macklin Chemical and used as received. Piperidine (99.5 %) was bought from SCR Chemical. TEB (97%) was supplied from Sigma Chemical. All aqueous solutions were prepared using deionized (DI) water with a resistivity of 18.2 MΩ·cm.

### Preparation of HsGDY@Cu_2_O NWs/CF photocathode

The HsGDY coated Cu_2_O NWs supported on a CF (HsGDY@Cu_2_O NWs/CF) photocathode was fabricated by three-step of electrochemical anodization, annealing, and the Glaser coupling reaction. The CF was anodized in an alkaline solution (3 M NaOH) for 30 min with anodic current density of 45 mA cm^−2^ to form Cu(OH)_2_ NWs/CF, following which the color of the CF changed from red orange with metallic luster to sky blue. The as-anodized nanowire was annealed in a tube furnace at 550 °C for 4 h under nitrogen atmosphere, where the Cu(OH)_2_ NWs/CF were converted into Cu_2_O NWs/CF and the color of the Cu_2_O NWs changed to brick red. Finally, the top layer of Cu_2_O was coved with HsGDY through a Glaser coupling reaction with TEB (5 mg, 0.033 mmol) as precursor. The piperidine (10 μL, 0.1 mmol) were added to a reaction tube with 10 mL pyridine as a solvent. Remarkably, the reaction tube is filled with oxygen. Cu_2_O NWs/CF was immersed in the reaction mixture and then the tube was sealed and heated in an oil bath pan to 60 °C for different durations. After reaction, the samples were immediately washed sequentially with pyridine, dichloromethane, and methanol. At last, the samples were blow-dried by dry nitrogen jet and the surface of the Cu_2_O NWs was covered with a golden yellow film. The HsGDY on copper foam has also prepared with the same method. The Pt/HsGDY@Cu_2_O NWs were also prepared through electrochemical deposition of platinum nanoparticles from a solution of 1 mM H_2_PtCl_6_ in deionized water at −0.1 V versus Ag/AgCl for 15 min.

### Materials characterizations

The morphologies of samples were characterized by scanning electron microscopy (SEM, S4800, Hitachi), and transmission electron microscopy (TEM, JEOLJEM 2100). The crystalline structure and chemical structure of the samples were analyzed by X-ray diffraction (XRD) (Bruker D8 Discover diffractometer, using Cu Kα radiation (1.540598 Å)) and DXR Raman Microscope (ThermoFisher Scientific) with excitation of 532 nm laser. The X-ray photoelectron spectroscopy (XPS) was carried out to reveal hybridization of elements, collected by an Axis Ultra instrument (Kratos Analytical) under ultrahigh vacuum (<10^−8^ torr) and using a monochromatic Al Kα X-ray source, with binding energies referenced to the C 1s binding energy of 284.8 eV. The diffuse reflectance UV-vis adsorption spectra were recorded on a spectrophotometer (Shimadzu, UV 3600), with the reference of BaSO_4_ powder. Photoluminescence (PL) emission spectra were measured using a photoluminescence spectrometer (FLS980, Edinburgh Instruments Ltd.) from 300 to 750 nm under an excitation wavelength of 260 nm. The electrochemical characterizations of electrochemical impedance spectra (EIS) and capacity were measured by a PGSTAT 302 N Autolab Potentiostat/Galvanostat (Metrohm) equipped with an excitation signal of 10 mV amplitude, and the Mott-Schottky measurement was performed at fixed frequency of 1 kHz.

### Photoelectrochemical measurements

All the PEC measurements were employed with CHI 660E electrochemical working station (Chenhua Instrument Co., Ltd., Shanghai), in a three-electrode system: the reference electrode was Ag/AgCl with saturated KCl solution, the counter electrode was a platinum foil, and the working electrode was the HsGDY@Cu_2_O NWs/CF photocathode. The supporting electrolyte was a 0.1 M Na_2_SO_4_ solution with pH to 4.9. The potential relation between reference electrode (Ag/AgCl with saturated KCl solution) and reversible hydrogen electrode (RHE) was calculated through the following equation:

2 $${{{{\rm{E}}}}}_{{{{\rm{RHE}}}}}={{{{\rm{E}}}}}_{{{{\rm{Ag}}}}/{{{\rm{AgCl}}}}}+0.059{{{\rm{pH}}}}+{{{\rm{E}}}}{^{\circ} _{{{{\rm{Ag}}}}/{{{\rm{AgCl}}}}}}$$

while $${{{\rm{E}}}}{^{\circ} _{{{{\rm{Ag}}}}/{{{\rm{AgCl}}}}}}=0.1976\,{{{\rm{V}}}}\,{{{\rm{at}}}}\,25^{\circ} {{{\rm{C}}}}$$.

Linear scanning voltammetry (LSV) was conducted with a slow scan rate of 5 mV s^−1^. The intensity of the light source was regulated with a Si diode (Model 818, Newport) to simulate AM 1.5 illumination (100 mW cm^−2^). The stability measurements of the photocathode were detected under 0 V vs RHE. The hydrogen generation reaction was performed under 0 V vs RHE in a gas-closed system equipped with a gas-circulated pump. The water-reduction gaseous products at different reaction time were analyzed on an online gas chromatograph (GC-9790II, FULI Corp., China) equipped with a flame ionization detector (FID) and a thermal conductivity detector (TCD). The PEC performance under illumination of concentrated solar light was also estimated. It was need to be noted that the concentrated solar light was filtered through cycling cool water, coated on the outer surface of PEC cells, before it reached the PEC setup to maintain the experimental temperature at 25 °C.

### DFT calculation

The DFT calculation were performed with the CP2K quantum chemistry software package, using Perdew–Burke–Ernzerhof (PBE) parametrization of generalized gradient approximation (GGA) to describe the exchange-correlation part in Hamiltonian^36^. We use GTH potential and Molopt basis set (DZVP-MOLOPT-SR-GTH) with an energy cutoff of 400 Ry^43^. Van der Waals (vdW) interaction was taken into account at the DFT-D3 level as proposed by Grimme^44^. Both atomic position and cell parameters were relaxed until the max force is <4.5 × 10^−4^ Ha/bohr.

## Supplementary information

Supplementary Inforamtion

## Data Availability

All data are available from the corresponding author upon reasonable request.
