# Supplementary material for: Hydrogen-substituted graphdiyne encapsulated cuprous oxide photocathode for efficient and stable photoelectrochemical water reduction
Source: Nat Commun. 2022 Oct 1;13:5770. doi: 10.1038/s41467-022-33445-z (PMC9526745; doi:10.1038/s41467-022-33445-z)
Supplement: Supplementary file 1 — Supplementary Inforamtion [file 41467_2022_33445_MOESM1_ESM.pdf]

Supplementary Information

Hydrogen-substituted graphdiyne encapsulated cuprous oxide photocathode for efficient and stable photoelectrochemical water reduction

Xue Zhou[1], Baihe Fu[1], Linjuan Li[1], Zheng Tian[1], Xiankui Xu[1], Zihao Wu[1,2], Jing Yang[1,3] & Zhonghai Zhang[1]✉

[1] Shanghai Key Laboratory of Green Chemistry and Chemical Processes, School of Chemistry and Molecular Engineering, East China Normal University, Shanghai 200241, China.

[2] Present addresses: Beijing National Laboratory for Molecular Sciences, College of Chemistry and Molecular Engineering, Peking University, Beijing 100871, China.

[3] Present addresses: Department of Chemistry, Fudan University, 2205 Songhu Road, Shanghai 200438, China.

✉e-mail: zhzhang@chem.ecnu.edu.cn

Table of contents of supplementary information:

Supplementary Fig. 1 EDX spectrum of HsDGY@Cu_2O NWs, the inset is the table of atomic ratios of Cu, O, and C.

Supplementary Fig. 2 XRD pattern of HsGDY@Cu_2O/CF.

Supplementary Fig. 3 XPS survey of Cu_2O NWs and HsGDY@Cu_2O NWs.

Supplementary Fig. 4 (a) LSV plots and (b) photocurrent densities at 0 V vs RHE of HsGDY@Cu_2O NWs prepared with different durations of Glaser coupling reactions; TEM images of HsGDY@Cu_2O NWs prepared with different durations of Glaser coupling reaction of (c) 6 h, (d) 9 h, and (e) 12 h, the scale bar is 100 nm.

Supplementary Fig. 5 LSV curve on HsGDY@Cu_2O NWs/CF photocathode with chopped light illumination.

Supplementary Fig. 6 (a) Cyclic voltammograms on HsGDY@Cu_2O NWs/CF with different scan rates and (b) the calculated capacitance on HsGDY@Cu_2O NWs/CF.

Supplementary Fig. 7. (a) LSV curves of HsGDY/copper foam in dark and under illumination of simulated solar light with AM 1.5G filter; (b) optical absorption spectrum of HsGDY after subtract optical of copper foam from HsGDY/copper foam; (c) hydrogen generation on HsGDY/copper foam at potential of 0 V vs RHE under illumination of simulated solar light (100 mW cm^{-2}, AM 1.5G); (d) IPCE plot of HsGDY/copper foam under a constant potential of 0 V vs RHE in wavelength region from 300 nm to 800 nm.

Supplementary Fig. 8 Integrated photocurrent densities with IPCE values under AM 1.5G simulated solar light (ASTM G173-03).

Supplementary Fig. 9 IPCE plots of HsGDY@Cu_2O NWs at different applied potentials of -0.4 V vs RHE and +0.4 V vs RHE.

Supplementary Fig. 10 SEM images of HsGDY@Cu_2O NWs/CF after (a) 80 h, (b) 100 h, and (c) 120 h stability measurements.

Supplementary Fig. 11 Core-level XPS of (a) C 1s, (b) Cu 2p, and (c) O 1s of HsGDY@Cu_2O NWs/CF after 80 h stability test; core-level XPS of (d) C 1s, (e) Cu 2p, and (f) O 1s of HsGDY@Cu_2O NWs/CF after 120 h stability test.

Supplementary Fig. 12 LSV curves of HsGDY@Cu_2O NWs and Pt/HsGDY@Cu_2O NWs in dark and under illumination.

Supplementary Fig. 13 Electrochemical impedance spectra of Cu_2O NWs and HsGDY@Cu_2O NWs in dark and under illumination.

Supplementary Fig. 14 Comparison of the photocurrent density on Cu_2O-based photocathodes for PEC water reduction.

Supplementary Table 1. Comparison of photoelectrochemical performances and stability on Cu_2O-based photocathodes.

Additional References

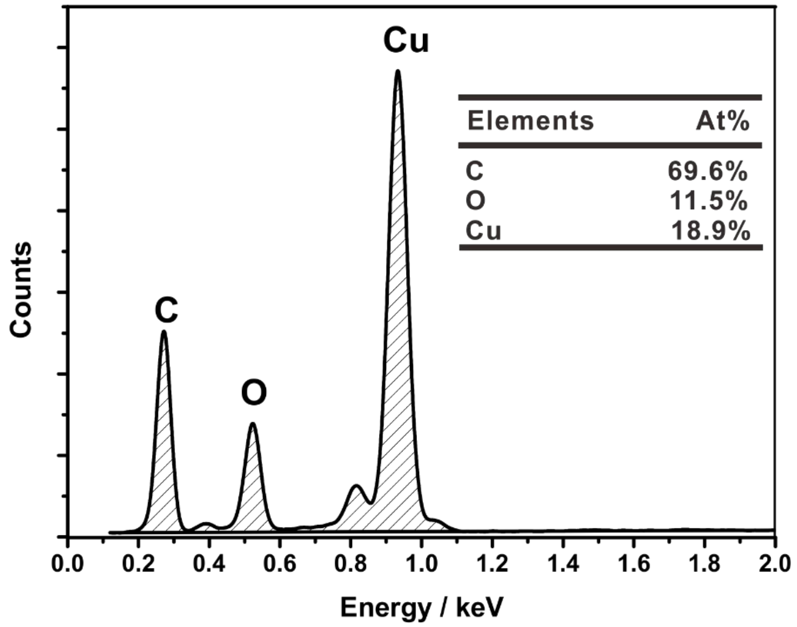

Supplementary Fig. 1 EDX spectrum of HsDGY@Cu_2O NWs, the inset is the table of atomic ratios of Cu, O, and C.

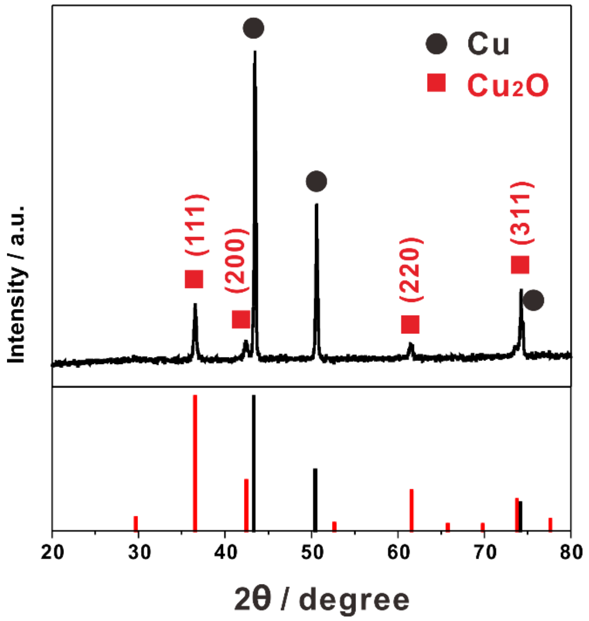

Supplementary Fig. 2 XRD pattern of HsGDY@Cu_2O/CF.

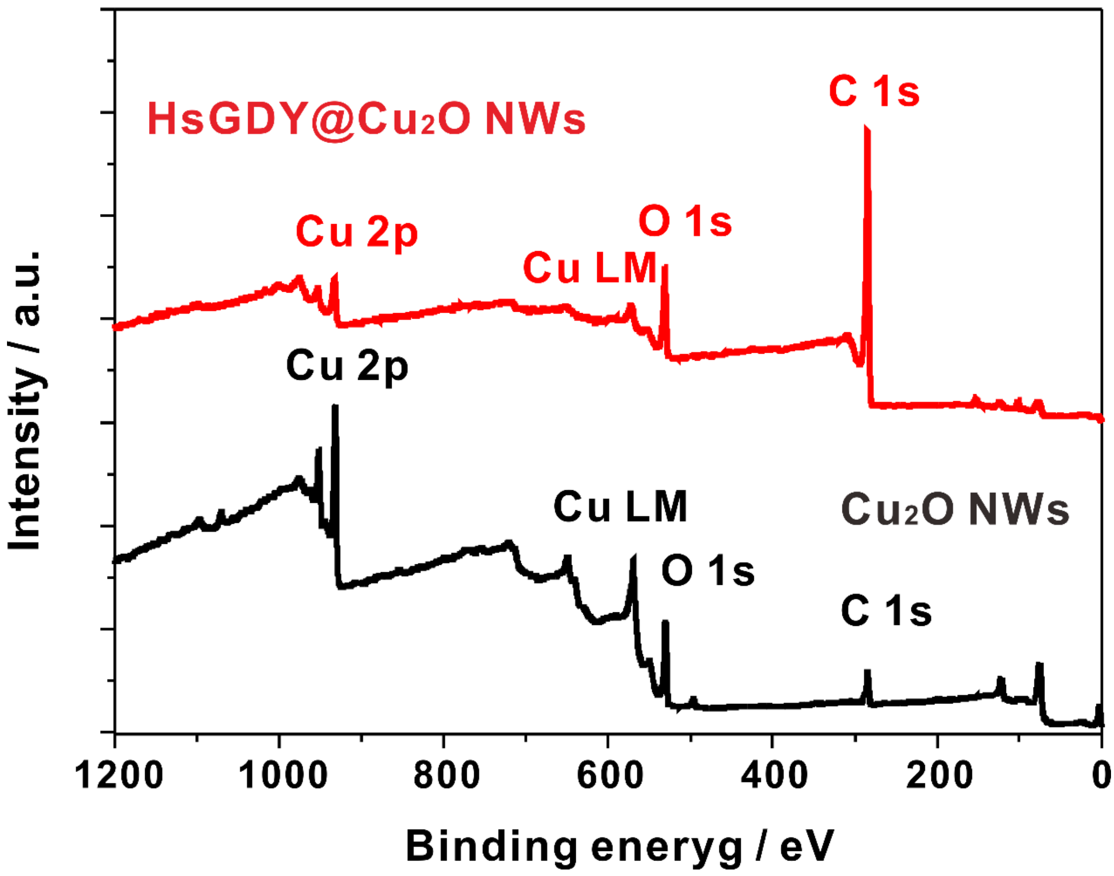

Supplementary Fig. 3 XPS survey of Cu_2O NWs and HsGDY@Cu_2O NWs.

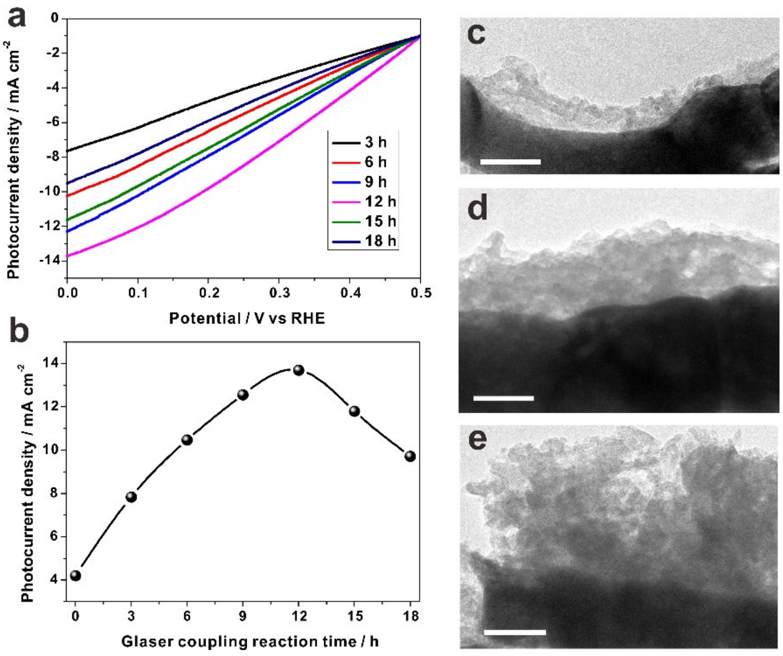

Supplementary Fig. 4 (a) LSV plots and (b) photocurrent densities at 0 V vs RHE of HsGDY@Cu_2O NWs prepared with different durations of Glaser coupling reactions; TEM images of HsGDY@Cu_2O NWs prepared with different durations of Glaser coupling reaction of (c) 6 h, (d) 9 h, and (e) 12 h, the scale bar is 100 nm.

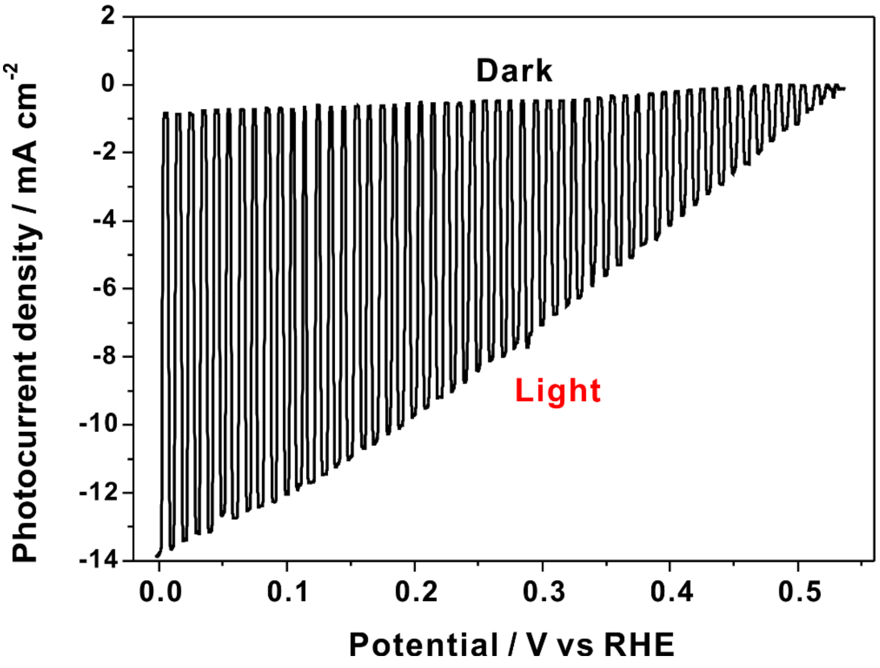

Supplementary Fig. 5 LSV curve on HsGDY@Cu_2O NWs/CF photocathode with chopped light illumination.

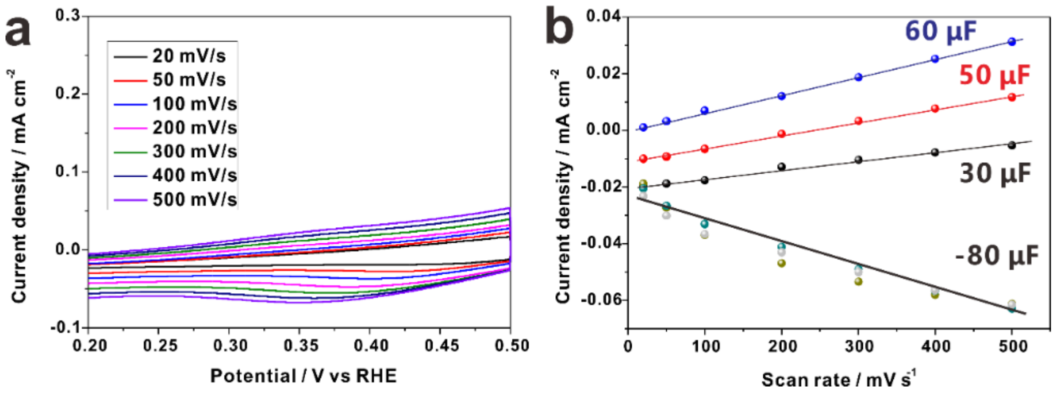

Supplementary Fig. 6 (a) Cyclic voltammograms on HsGDY@Cu_2O NWs/CF with different scan rates and (b) the calculated capacitance on HsGDY@Cu_2O NWs/CF.

The electrochemical active surface area measurements were performed through Helmholtz double layer capacitance measurements following P. Connor and co-authors proposed method (P. Connor, J. Schuch, B. Kaiser, W. Jaegermann, The Determination of Electrochemical Active Surface Area and Specific Capacity Revisited for the System MnOx as an Oxygen Evolution Catalyst, Z. Phys. Chem. 2020; 234(5): 979–994). As

presented in Supplementary Fig. 6, capacitance of HsGDY@Cu_2O NWs/CF can be calculated from the non-faradaic current values as the following equations:

$$ECSA = C_{DL}/C_S \quad (1)$$

$$C_{DL} = dQ/d\varphi \quad (2)$$

$$i = dQ(\varphi)/dt \quad (3)$$

with ECSA as electrochemical active surface area, C_{DL} as the double layer capacitance, C_S as the specific capacitance, Q as charge, φ as potential, i as current, and t as time. The average value of 40 μF cm^{-2}, reported by McCrory (J. Am. Chem. Soc. 2015, 137, 4347) was used as value for the specific capacitance (C_S). The C_{DL} of different representative potential was calculated and the average electrochemical active surface area can be measured to be 1.38 ± 0.63 cm^2. Therefore, the intrinsic photocurrent density of HsGDY@Cu_2O NWs/CF can be converted to be 9.33 ± 2.92 mA cm^{-2}.

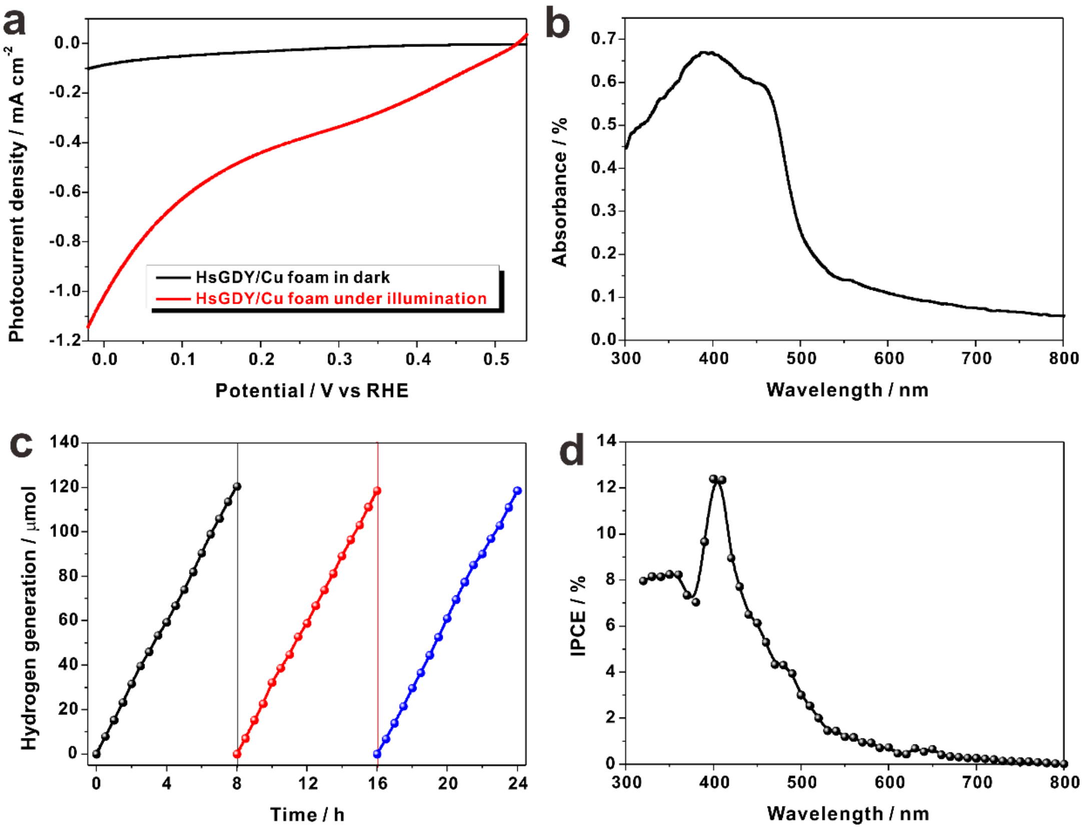

Supplementary Fig. 7. (a) LSV curves of HsGDY/copper foam in dark and under illumination of simulated solar light with AM 1.5G filter; (b) optical absorption spectrum of HsGDY after subtract optical of copper foam from HsGDY/copper foam; (c) hydrogen generation on HsGDY/copper foam at potential of 0 V vs RHE under illumination of simulated solar light (100 mW cm^{-2}, AM 1.5G); (d) IPCE plot of HsGDY/copper foam under a constant potential of 0 V vs RHE in wavelength region from 300 nm to 800 nm.

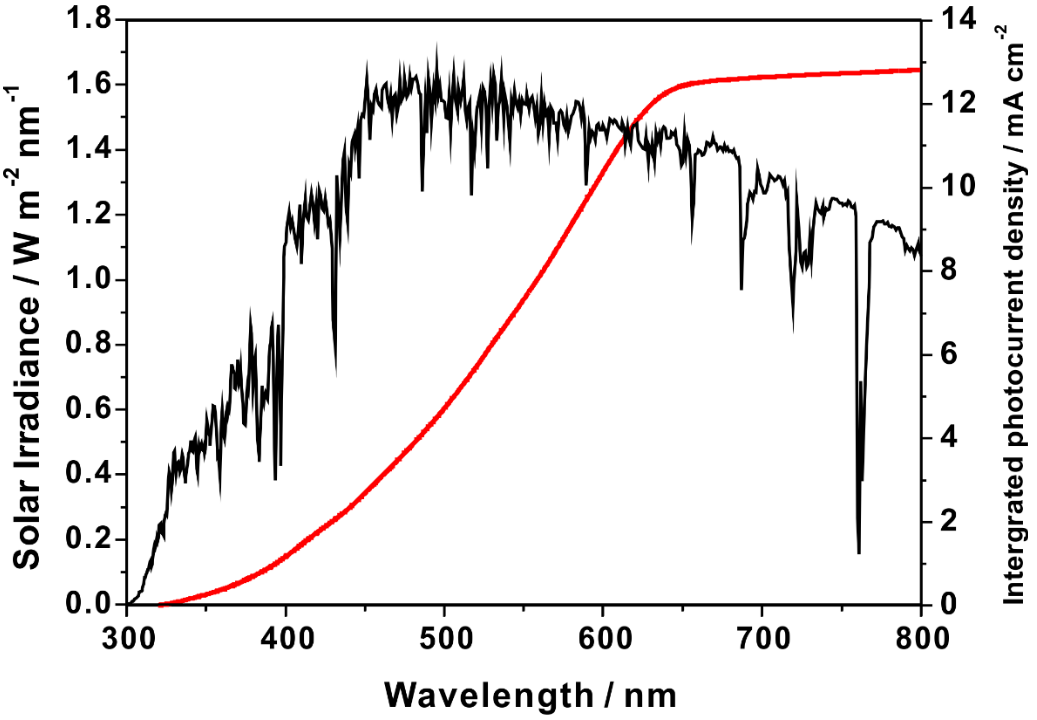

Supplementary Fig. 8 Integrated photocurrent densities with IPCE values under AM 1.5G simulated solar light (ASTM G173-03).

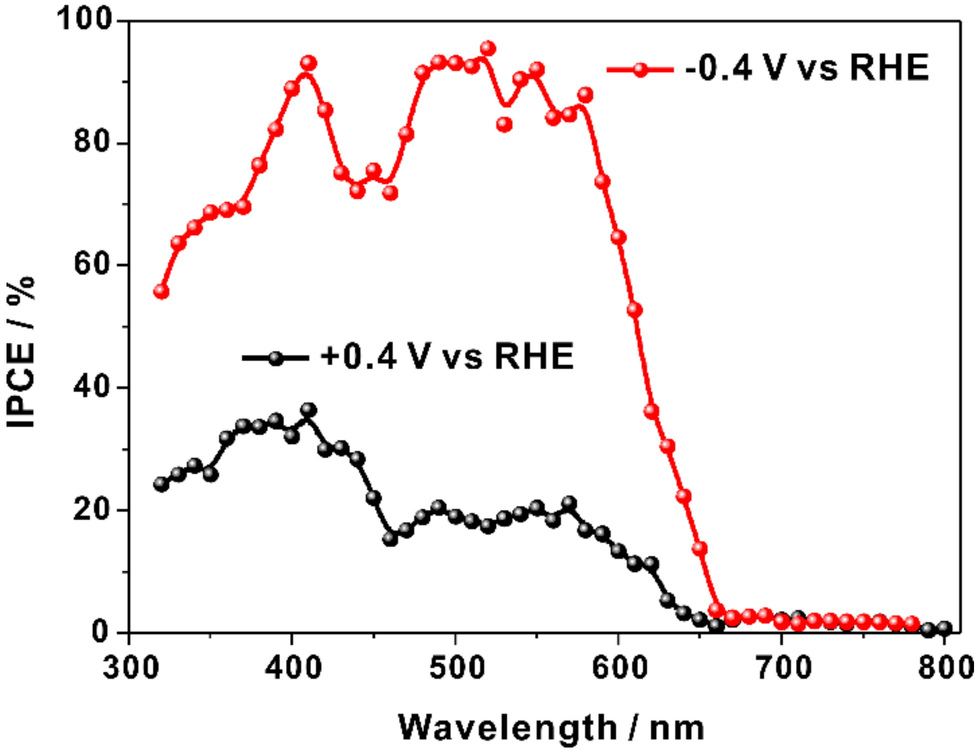

Supplementary Fig. 9 IPCE plots of HsGDY@Cu_2O NWs at different applied potentials of -0.4 V vs RHE and +0.4 V vs RHE.

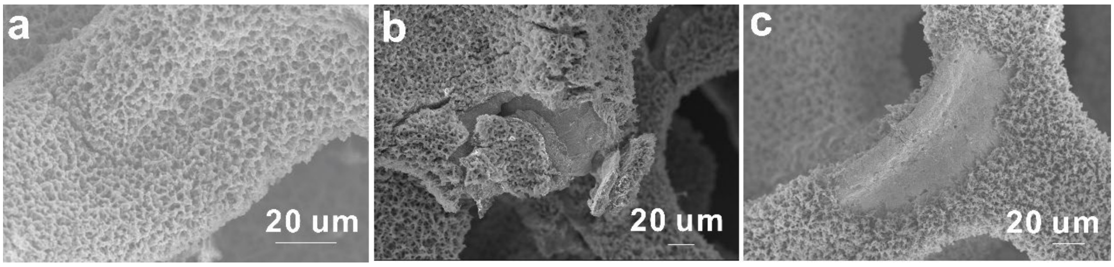

Supplementary Fig. 10 SEM images of HsGDY@Cu_2O NWs/CF after (a) 80 h, (b) 100 h, and (c) 120 h stability measurements.

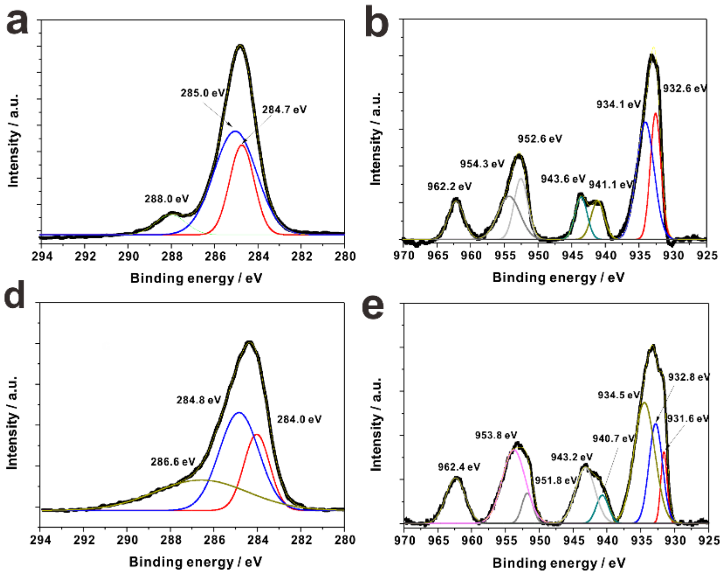

Supplementary Fig. 11 Core-level XPS of (a) C 1s, (b) Cu 2p, and (c) O 1s of HsGDY@Cu_2O NWs/CF after 80 h stability test; core-level XPS of (d) C 1s, (e) Cu 2p, and (f) O 1s of HsGDY@Cu_2O NWs/CF after 120 h stability test.

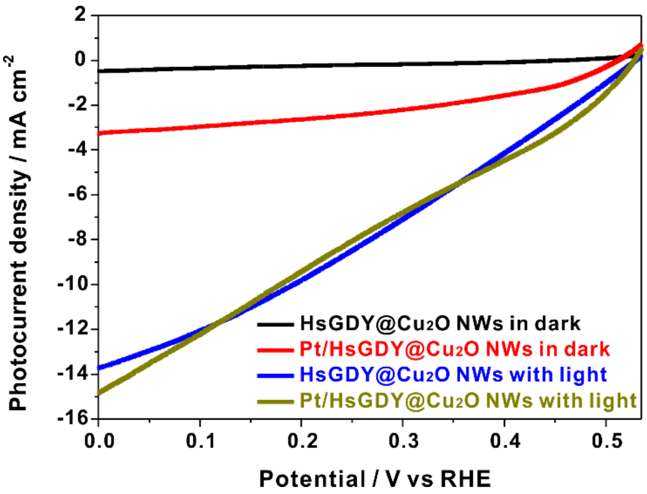

Supplementary Fig. 12 LSV curves of HsGDY@Cu_2O NWs and Pt/HsGDY@Cu_2O NWs in dark and under illumination.

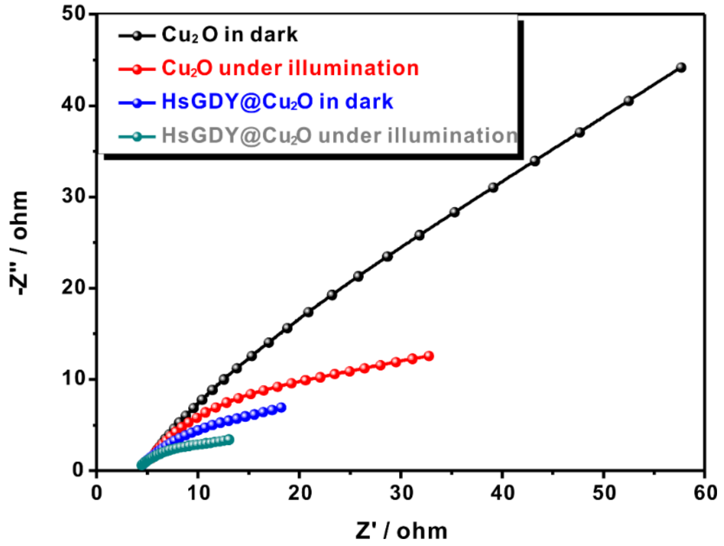

Supplementary Fig. 13 Electrochemical impedance spectra of Cu_2O NWs and HsGDY@Cu_2O NWs in dark and under illumination.

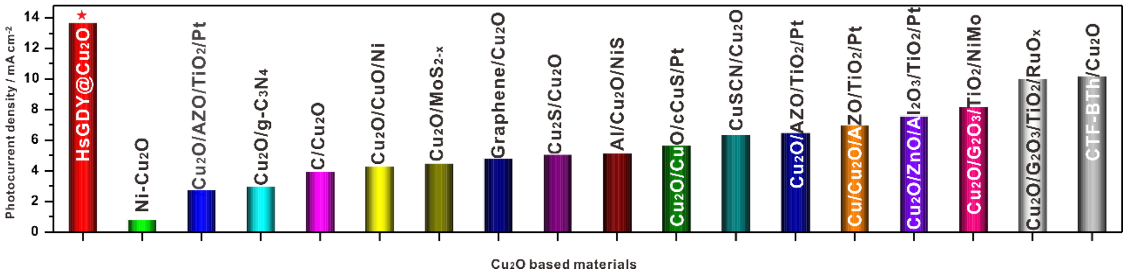

Supplementary Fig. 14 Comparison of the photocurrent density on Cu_2O-based photocathodes for PEC water reduction.

Supplementary Table 1. Comparison of photoelectrochemical performances and stability on Cu_2O-based photocathodes.

Photocathodes	mA cm^{-2} at 0 V vs RHE	Stability for 2 h	reference
Ni-Cu_2O	0.83	90.2% 2 h	1
$Cu_2O/Ga_2O_3/TiO_2$/RuOx	10	/	2
$Cu_2O/Ga_2O_3/TiO_2$/NiMo	8.2	90% 8 h	2
CuSCN/Cu_2O	6.4	94% 60 h	3
CTF-BTh/Cu_2O	10.2	/	4
C/Cu_2O NWs	3.96	80.7 20 min	5
Cu_2O/CuO/Ni	4.3	87.7 20 min	6
Cu_2O/MoS_{2+x}	4.5	/	7
Graphene/Cu_2O NWs	4.8	83% 20 min	8
Cu_{2S}/Cu_2O NWs	5.05	/	9
Cu_2O/CuO/CuS/Pt	5.7	92% 1 h	10
Cu_2O/AZO/TiO_2/Pt	6.5	/	11
Cu/Cu_2O/AZO/TiO_2/Pt	7	75% 20 min	12
$Cu_2O/ZnO/Al_2O_3/TiO_2$/Pt	7.6	33% 20 min	13
Al/Cu_2O/NiS	5.16	/	14
Cu_2O foam/g-C_3N_4	3	64% 30 min	15
**HsGDY@Cu_2O NWs**	**13.7**	**92.5% after 24 h**	**This work**

Additional References:

1. Zhang, M., Wang, J., Xue, H., Zhang, J., Peng, S., Han, X., Deng, Y. & Hu, W. Acceptor-Doping Accelerated Charge Separation in Cu_2O Photocathode for Photoelectrochemical Water Splitting: Theoretical and Experimental Studies. *Angew. Chem. Int. Ed.* **59**, 18463–18467 (2020).
2. Pan, L., Kim, J. H., Mayer, M. T., Son2, M. K., Ummadisingu, A., Lee, J. S., Hagfeldt, A., Luo, J. & Grätzel, M. Boosting the performance of Cu_2O photocathodes for unassisted solar water splitting devices. *Nat. Catal.* **1**, 412-420 (2018).
3. Pan, L., Liu, Y., Yao, L., Ren, D., Sivula, K., Grätzel, M. & Hagfeldt, A. Cu_2O photocathodes with band-tail states assisted hole transport for standalone solar water splitting. *Nat. Commun.* **11**, 318 (2020).
4. Zhang, Y., Lv, H., Zhang, Z., Wang, L., Wu, X. & Xu, H. Stable Unbiased Photo-Electrochemical Overall Water Splitting Exceeding 3% Efficiency via Covalent Triazine Framework/Metal Oxide Hybrid Photoelectrodes. *Adv. Mater.* **33**, 2008264 (2021).
5. Zhang, Z., Dua, R., Zhang, L., Zhu, H., Zhang, H. & Wang, P. Carbon-Layer-Protected Cuprous Oxide Nanowire Arrays for Efficient Water Reduction. *ACS Nano* **7**, 1709-1717 (2013).
6. Dubale, A. A., Pan, C. J., Tamirat, A. G., Chen, H. M., Su, W. N., Chen, C. H., Rick, J., Ayele, D. W., Aragaw, B. A., Lee, J. F., Yang, Y. W., Hwang, B. J. Heterostructured Cu_2O/CuO decorated with nickel as a highly efficient

photocathode for photoelectrochemical water reduction. *J. Mater. Chem. A* 2015, **3**, 12482-12499.

7. Morales-Guio, C. G., Tilley, S. D., Vrubel, H., Grätzel, M. & Hu, X. Hydrogen evolution from a copper(I) oxide photocathode coated with an amorphous molybdenum sulphide catalyst. *Nat. Commun*. **5**, 3059 (2014).
8. Dubale, A. A., Su, W. N., Tamirat, A. G., Pan, C. J., Aragaw, B. A., Chen, H. M., Chen, C. H. & Hwang, B. J. The synergetic effect of graphene on Cu_2O nanowire arrays as a highly efficient hydrogen evolution photocathode in water splitting. *J. Mater. Chem. A* **2**, 18383-18397 (2014).
9. Li, Z. & Zhang, Z. Tetrafunctional Cu_2S thin layers on Cu_2O nanowires for efficient photoelectrochemical water splitting. *Nano Res* **11**, 1530-1540 (2018).
10. Dubale, A. A., Tamirat, A. G., Chen, H. M., Berhe, T. A., Pan, C. J., Su, W. N. & Hwang, B. J. A highly stable CuS and CuS–Pt modified Cu_2O/CuO heterostructure as an efficient photocathode for the hydrogen evolution reaction. *J. Mater. Chem. A* **4**, 2205-2216 (2016).
11. Paracchino, A., Mathews, N., Hisatomi, T., Stefik, M., Tilley, S. D. & Grätzel, M. Ultrathin films on copper(I) oxide water splitting photocathodes: a study on performance and stability. *Energy Environ. Sci.* **5**, 8673-8681 (2012).
12. Jin, Z., Hu, Z., Yu, J. C. & Wang, J. Room temperature synthesis of a highly active Cu/Cu_2O photocathode for photoelectrochemical water splitting. *J. Mater. Chem. A* **4**, 13736-13741 (2016).
13. Paracchino, A. Laporte, V., Sivula, K., Grätzel, M. and Thimsen. E. Highly active oxide photocathode for photoelectrochemical water reduction. *Nat. Mater*. **10**, 456-461 (2011).
14. Chen, D., Liu, Z., Guo, Z., Yan, W. & Ruan, M. Decorating Cu_2O photocathode with noble-metal-free Al and NiS cocatalysts for efficient photoelectrochemical water splitting by light harvesting management and charge separation design. *Chem. Eng. J.* **381**, 122655 (2020).
15. Ma, X., Zhang, J., Wang, B., Li, Q. & Chu, S. Hierarchical Cu_2O foam/g-C_3N_4 photocathode for photoelectrochemical hydrogen production. *Appl. Surf. Sci.* **427**, 907-916 (2018).
